# GPR4 Knockout Improves the Neurotoxin-Induced, Caspase-Dependent Mitochondrial Apoptosis of the Dopaminergic Neuronal Cell

**DOI:** 10.3390/ijms21207517

**Published:** 2020-10-12

**Authors:** Md Ezazul Haque, Mahbuba Akther, Shofiul Azam, Dong-Kug Choi, In-Su Kim

**Affiliations:** 1 Department of Applied Life Science, Graduate School, Konkuk University, Chungju 27478, Korea; mdezazulhaque@yahoo.com (M.E.H.); smritymahbuba@gmail.com (M.A.); shofiul_azam@hotmail.com (S.A.); choidk@kku.ac.kr (D.-K.C.); 2 Department of Biotechnology, Research Institute of Inflammatory Disease (RID), College of Biomedical and Health Science, Konkuk University, Chungju 27478, Korea

**Keywords:** apoptosis, neurodegeneration, GPR4 receptor, MPP^+^, Parkinson’s disease, CRISPR/cas9

## Abstract

In Parkinson’s disease, mitochondrial oxidative stress-mediated apoptosis is a major cause of dopaminergic neuronal loss in the substantia nigra (SN). G protein-coupled receptor 4 (GPR4), previously recognised as an orphan G protein coupled-receptor (GPCR), has recently been claimed as a member of the group of proton-activated GPCRs. Its activity in neuronal apoptosis, however, remains undefined. In this study, we investigated the role of GPR4 in the 1-methyl-4-phenylpyridinium ion (MPP^+^) and hydrogen peroxide (H_2_O_2_)-treated apoptotic cell death of stably GPR4-overexpressing and stably GPR4-knockout human neuroblastoma SH-SY5Y cells. In GPR4-OE cells, MPP^+^ and H_2_O_2_ were found to significantly increase the expression levels of both mRNA and proteins of the pro-apoptotic Bcl-2-associated X protein (Bax) genes, while they decreased the anti-apoptotic B-cell lymphoma 2 (Bcl-2) genes. In addition, MPP^+^ treatment activated Caspase-3, leading to the cleavage of poly (ADP-ribose) polymerase (PARP) and decreasing the mitochondrial membrane potential (ΔΨm) in GPR4-OE cells. In contrast, H_2_O_2_ treatment significantly increased the intracellular calcium ions (Ca^2+^) and reactive oxygen species (ROS) in GPR4-OE cells. Further, chemical inhibition by NE52-QQ57, a selective antagonist of GPR4, and knockout of GPR4 by clustered regularly interspaced short palindromic repeats (CRISPR)/Cas9 decreased the Bax/Bcl-2 ratio and ROS generation, and stabilised the ΔΨm, thus protecting the SH-SY5Y cells from MPP^+^- or H_2_O_2_-induced apoptotic cell death. Moreover, the knockout of GPR4 decreased the proteolytic degradation of phosphatidylinositol biphosphate (PIP_2_) and subsequent release of the endoplasmic reticulum (ER)-stored Ca^2+^ in the cytosol. Our results suggest that the pharmacological inhibition or genetic deletion of GPR4 improves the neurotoxin-induced caspase-dependent mitochondrial apoptotic pathway, possibly through the modulation of PIP_2_ degradation-mediated calcium signalling. Therefore, GPR4 presents a potential therapeutic target for neurodegenerative disorders such as Parkinson’s disease.

## 1. Introduction

Parkinson’s disease (PD) is a neurodegenerative disorder characterised by dopamine deficiency. An important pathological basis of PD is the loss of dopaminergic neurons due to apoptotic cell death in the substantia nigra (SN) of the brain [1]. An array of evidence suggests that reactive oxygen species (ROS)-induced oxidative stress is a major cause of the dopaminergic neuronal loss in the SN [2]. Mitochondria are key players in apoptosis during this neurodegeneration. In a cell undergoing apoptosis, mitochondria increase the production of oxyradicals and open the pores of its membranes, leading to the depolarisation of its transmembrane potential (Δψm) and the release of cytochrome C [3,4]. The B-cell lymphoma 2 (Bcl-2) family of proteins, which includes anti-apoptotic Bcl-2 and pro-apoptotic Bcl-2-associated X protein (Bax) members, plays a critical role in the initiation of the apoptotic pathway. When anti-apoptotic Bcl-2 localises in the mitochondrial inner membrane from the outer membrane, the intermembrane space protein, cytochrome C, is released. This binds to apoptotic peptidase activating factor 1 (APAF1) and forms a heptameric structure, known as the apoptosome. Apoptosomes recruit caspase 9 and activate a series of events, eventually activating Caspase-3 as a result [5]. Surprisingly, poly (ADP-ribose) polymerase (PARP), a nuclear protein involved in DNA repair that is specifically cleaved to a signature 89-kDa fragment, has been implicated as an early marker for apoptotic cell death in neurons [6]. The characteristic hallmarks of the mitochondrial oxidative damage-induced apoptosis pathway can be considered to be the opening of the mitochondrial permeability transition pore (mPTP), the collapse of the mitochondrial membrane potential (ΔΨm), the release of cytochrome C, the activation of Caspase-3, and proteolytic degradation of PARP [7,8]. Surprisingly, the endoplasmic reticulum (ER) acts as a reservoir of calcium ions (Ca^2+^). The ER can release Ca^2+^ through either its ryanodine (RYR) or inositol trisphosphate (IP3) receptors. Ca^2+^, once released from the ER, is taken up by the mitochondrial Ca^2+^ uniporter (MCU) located in the mitochondrial inner membrane [9]. An increase in mitochondrial Ca^2+^ is associated with an increase of the mPTP opening and ROS generation, a decrease in ΔΨm, and release of cytochrome C, as well as excitotoxicity and apoptosis [10,11,12]. Recent findings suggest that an increase in the Ca^2+^ released from the ER can increase the mitochondrial oxidant stress of the substantia nigra pars compacta (SNc) dopaminergic (DA) neurons [10,13]. Alteration of the intracellular calcium homeostasis PD cybrids has also been reported [14].

1-methyl-4-phenylpyridinium ion (MPP^+^) and hydrogen peroxide (H_2_O_2_) are the most widely employed neurotoxins, due to their ability to mimic a PD-like syndrome with apoptotic cell death through mitochondrial oxidative damage, in both cellular and animal models of PD [15,16,17]. MPP^+^, the active metabolite of 1-methyl-4-phenyl-1,2,3,6-tetrahydropyridine (MPTP), generates 5–7 times more ROS by selectively inhibiting mitochondrial complex I, thus initiating pro-apoptotic Bax/Bcl-2-dependent apoptotic cell death [15]. Besides, MPP^+^- and H_2_O_2_-mediated oxidative stress have also been reported to cause mitochondrial oxidative stress-mediated apoptotic cell death [18]. Moreover, MPP^+^- and H_2_O_2_-mediated cell death are associated with the alteration of intracellular Ca^2+^ homeostasis. An increase in intracellular Ca^2+^ due to ER stress also potentiates a decrease in ΔΨm, the release of cytochrome C, and the induction of apoptosis [12,19,20,21]. Therefore, the inhibition of pro-apoptotic signalling, or the decrease of intracellular ROS and Ca^2+^, may be beneficial for the protection of dopaminergic neuronal loss in PD.

G protein-coupled receptor 4 (GPR4), a proton-sensing receptor, is highly sensitive to the alteration of extracellular proton concentration [22]. It belongs to a small G protein-coupled proton-sensing receptor family that includes ovarian cancer G protein-coupled receptor 1 (OGR1), also referred to as GPR68, G2A, also termed GPR132, and T-cell death-associated gene 8 (TDAG8), also known as GPR65. These receptors signal through either to phosphoinositide mediated increase in intracellular Ca^2+^ or through modulating adenylate cyclase activity [23,24]. Information regarding the distribution and biology of GPR4 in the brain of an individual with PD is limited. An abundant level of GPR4 expression was observed in the retro-trapezoidal nucleus locus coeruleus, the cerebrovascular endothelium, the neurons of the dorsal raphe, and the lateral septum of a GPR4-knock-in mouse model [25]. Recently, the role of GPR4 in inflammation during ER stress pathway-mediated apoptotic cell death has been reported. In human umbilical vein endothelial cells (HUVEC) and other disease models, such as that of myocardial ischemic mice, knocking out GPR4 has been found to reduce cardiomyocyte apoptosis and improve cardiac function [26,27,28]. However, no study has yet to elucidate the role of GPR4 on apoptotic cell death in neurodegenerative disorders. Therefore, this work aims to forge an understanding of the role of GPR4 in neurotoxin-induced, mitochondrial oxidative stress-mediated apoptosis in a PD model. In particular, through our study of the overexpression and genetic deletion of GPR4, we investigate the role of GPR4 in the mitochondrial apoptosis pathway.

## 2. Results

### 2.1. Expression of GPR4 is Upregulated in Neurotoxin-Stimulated Apoptosis in SH-SY5Y Cells

**Figure 1:**
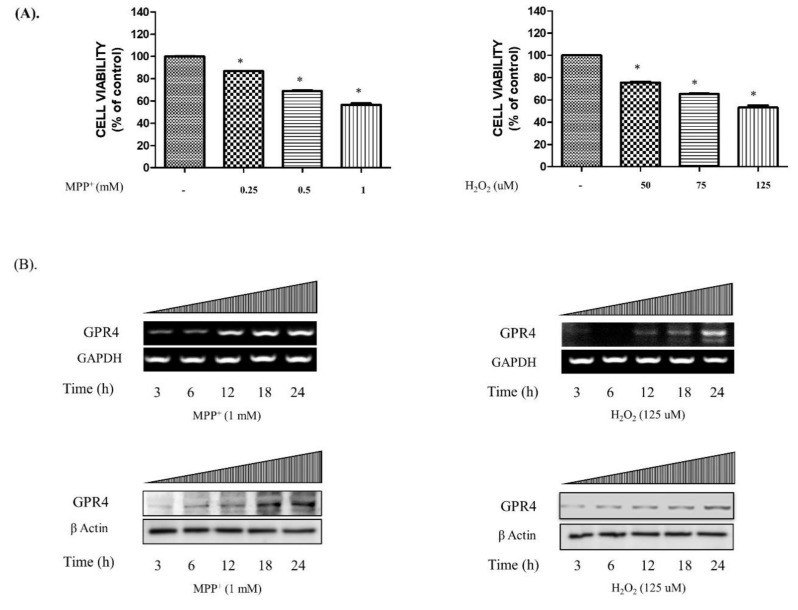
The cellular viability and the mRNA and protein expressions of GPR4 in MPP^+^- and H_2_O_2_-treated SH-SY5Y cells. 24 h serum-starved SH-SY5Y cells were treated with different concentrations of MPP^+^ (0.25, 0.5, and 1 mM) and H_2_O_2_ (50, 75, and 125 µM) for 24 h in serum-free culture media. (**A**) The cellular viability of the SH-SY5Y cell after treatment with different concentrations of MPP^+^ and H_2_O_2_ in serum-free media for 24 h. (**B**) Reverse transcription-polymerase chain reaction (RT-PCR) and immunoblotting demonstrate the mRNA and protein expressions of GPR4 in SH-SY5Y cells at different time points (3, 6, 12, 18, and 24 h) after stimulation with MPP^+^ (1 mM) and H_2_O_2_ (125 µM) in serum-free media. Glyceraldehyde-3-phosphate dehydrogenase (GAPDH) and β-actin were utilised as the internal controls. Mean ± standard error of the mean (SEM; *n* = 3) was employed to express the data. Tukey’s multiple comparison test was performed using a one-way analysis of variance (ANOVA). Each * *p* < 0.05 refers to the other sample concentrations compared with the control cells.

To investigate the concentrations of MPP^+^ and H_2_O_2_ that precipitated a cell death of nearly 50% in the SH-SY5Y cells, 24 h serum-starved SH-SY5Y cells were treated with MPP^+^ (0.25, 0.5, and 1 mM) or H_2_O_2_ (50, 75, and 125 µM) for 24 h. As is shown in Figure 1A, when treated with the various concentrations of MPP^+^ (1 mM; 56.511 ± 1.55%) and H_2_O_2_ (125 µM; 53.12 ± 2.34%), half of the cell population in the MTT assay died. Furthermore, the mRNA and protein expressions of GPR4 in SH-SY5Y cells in both MPP^+^- (1 mM) and H_2_O_2_- (125 µM) treated serum-free media gradually increased in a time-dependent manner (3–24 h; Figure 1B).

### 2.2. Knockout of GPR4 Protects SH-SY5Y Cells from Neurotoxin-Stimulated Apoptosis in SH-SY5Y Cells

**Figure 2:**
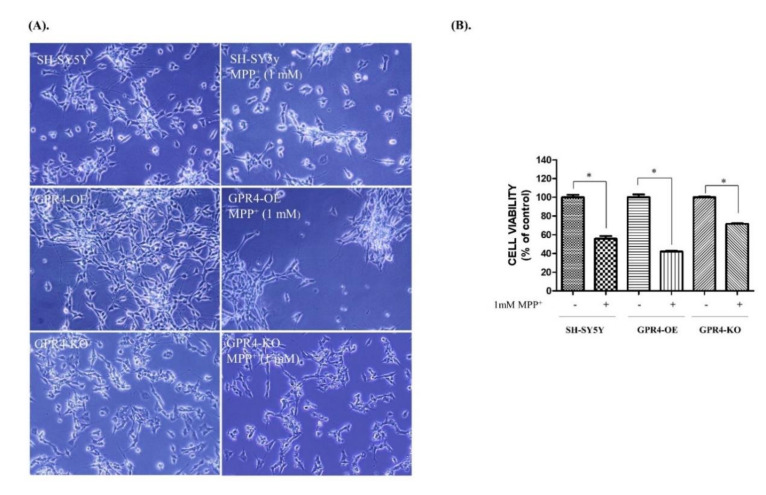
The cellular viability and morphology of MPP^+^-treated SH-SY5Y cells that were stably GPR4-overexpressing (GPR4-OE) or GPR4-knockout (GPR4-KO). 24 h serum-starved SH-SY5Y cells were treated with MPP^+^ (1 mM) for 24 h in serum-free culture media. (**A**) The morphology of SH-SY5Y GPR4-OE and GPR4-KO cells was observed through bright-field microscopy. (**B**) Cell viability was evaluated using an MTT assay. Mean ± SEM (*n* = 3) was employed to express the data. Tukey’s multiple comparison test was performed using a one-way ANOVA. Each * *p* < 0.05 refers to the other sample concentrations compared with the control cells.

To assess the effect of GPR4 overexpression and knockout on MPP^+^-induced apoptotic cell death, 24 h serum-starved SH-SY5Y cells were treated with MPP^+^ (1 mM) for 24 h in serum-free media (Figure 2). Following the MPP^+^ (1 mM) treatment for 24 h in serum-free media, the number of SH-SY5Y viable cells decreased. Furthermore, the cells became rounded, displayed an increased neurite retraction, and were found to be loosely attached to the plate. Under bright-field optics, the GPR4-OE cells treated with MPP^+^ (1 mM) exhibited less cell viability, with increased rounded cells, increased neurite retraction, and loose attachment to the surface. In contrast, the GPR4-KO cells treated with MPP^+^ (1 mM) were more viable, strongly attached, neuronal shaped, and demonstrated less neuronal retraction than both the control and the GPR4-OE cells (Figure 2A).

Cell viability was assessed with an MTT assay. The control SH-SY5Y cells presented a 55.67 ± 5.22% cell survival rate, whereas only 42.00 ± 2.01% of the GPR4-OE cells treated with MPP^+^ (1 mM) survived. In contrast, the MPP^+^-treated GPR4-KO cells had a significantly higher cell survival rate (71.63 ± 3.54%), at 15% higher than for the MPP^+^-treated control SH-SY5Y cells and almost 30% higher than for the MPP^+^-treated GPR4-OE cells (Figure 2B).

### 2.3. Knockout of GPR4 Decreases the Bax/Bcl-2 mRNA Ratio during Neurotoxin-Induced Apoptosis in SH-SY5Y Cells

**Figure 3:**
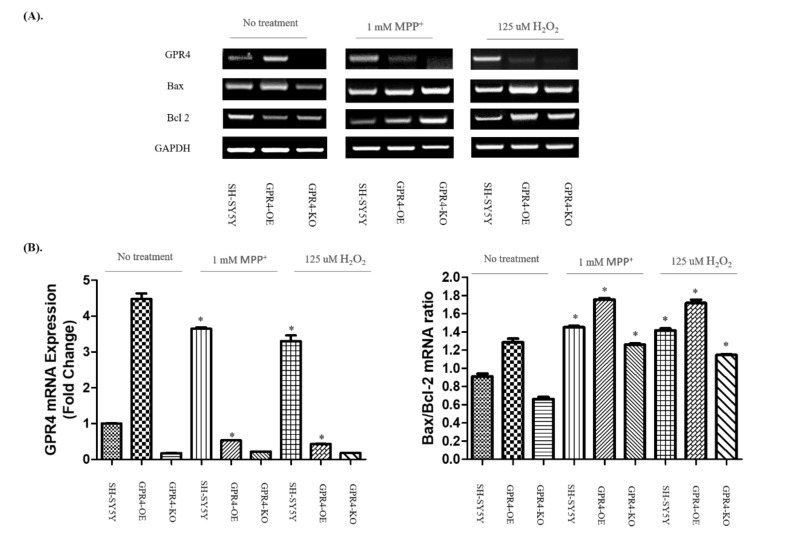
The measurement of GPR4 mRNA expression and the Bax/Bcl-2 mRNA ratio in MPP^+^- and H_2_O_2_-treated SH-SY5Y cells that were stably GPR4-OE or GPR4-KO. (**A**) An RT-PCR illustrating the mRNA expression of pro-apoptotic Bax, anti-apoptotic Bcl-2, and GAPDH in SH-SY5Y, GPR4-OE, and GPR4-KO cells after stimulation with MPP^+^ (1 mM) and H_2_O_2_ (125 µM) in serum-free media for 24 h. (**B**) A semi-quantification of the GPR4 mRNA and Bax/Bcl-2 mRNA expressions relative to GAPDH. This semi-quantification of the respective mRNA expression levels was performed on ImageJ software; GAPDH was utilised as an internal control. Mean ± SEM (*n* = 3) was employed to express the data. Tukey’s multiple comparison test was performed using a one-way ANOVA. Each * *p* < 0.05 refers to the sample concentration compared with the same group of non-treated cells.

To determine the role of GPR4 in both MPP^+^- (1 mM) and H_2_O_2_- (125 µM) stimulated apoptotic cell death, we investigated the expression levels of the Bcl-2 family proteins (Bax and Bcl-2). Many studies suggest that the Bcl-2 family plays a critical role in the mitochondrial apoptotic pathway. Bax enhances the release of cytochrome C from the space of the mitochondrial intermembrane to the cytosol, resulting in apoptosis. In contrast, Bcl-2 prevents apoptosis through its prevention of cytochrome C release, thereby maintaining mitochondrial cellular integrity [29,30]. In this study, an RT-PCR was employed to assess the mRNA expression levels of GPR4, Bax, and Bcl-2 in 24 h serum-starved SH-SY5Y cells treated with either MPP^+^ (1 mM) or H_2_O_2_ (125 µM; Figure 3A).

A semiquantitative analysis (Figure 3B) of the RT-PCR bands highlighted a more than 4-fold increase in the expression of GPR4 in the GPR4-OE cells without any treatment, compared with the non-treated SH-SY5Y cells. In comparison with the non-treated SH-SY5Y cells, neurotoxins increased the expression of GPR4 in the GPR4-OE cells by 3–4-fold (MPP^+^, 3.65 ± 0.03; H_2_O_2_, 3.31 ± 0.17), whereas no significant difference in the GPR4 expression of the GPR4-OE cells (MPP^+^, 0.53 ± 0.003; H_2_O_2_, 0.04 ± 0.003) was observed.

Interestingly, the ratio of Bax/Bcl-2 mRNA expression for non-treated GPR4-OE cells was slightly higher (1.40 ± 0.08) than that for the control SH-SY5Y cells, whereas the Bax/Bcl-2 mRNA ratio was slightly lower (0.73 ± 0.904) in the non-treated GPR4-KO group than that for the control SH-SY5Y cells. For the SHSY-5Y cells, treatment with MPP^+^ or H_2_O_2_ significantly increased the ratio of Bax/Bcl-2 mRNA expression (MPP^+^, 1.59 ± 0.02; H_2_O_2_, 1.55 ± 0.04). Yet, the ratio of Bax/Bcl-2 mRNA expression in MPP^+^- and H_2_O_2_-stimulated GPR4-OE cells was significantly higher (MPP^+^, 1.92 ± 0.03; H_2_O_2_, 1.88 ± 0.07) than the neurotoxin-treated SH-SY5Y and non-treated GPR4-OE cells. Meanwhile, the ratio of Bax/Bcl-2 mRNA expression in both MPP^+^- and H_2_O_2_-stimulated GPR4-KO cells was significantly lower (MPP^+^, 1.38 ± 0.02; H_2_O_2_, 1.25 ± 0.02) than both the neurotoxin-treated SH-SY5Y and GPR4-OE cells.

### 2.4. Effect of a GPR4 Antagonist on the Cellular Morphology and GPR4 mRNA Expression of SH-SY5Y Cells

**Figure 4:**
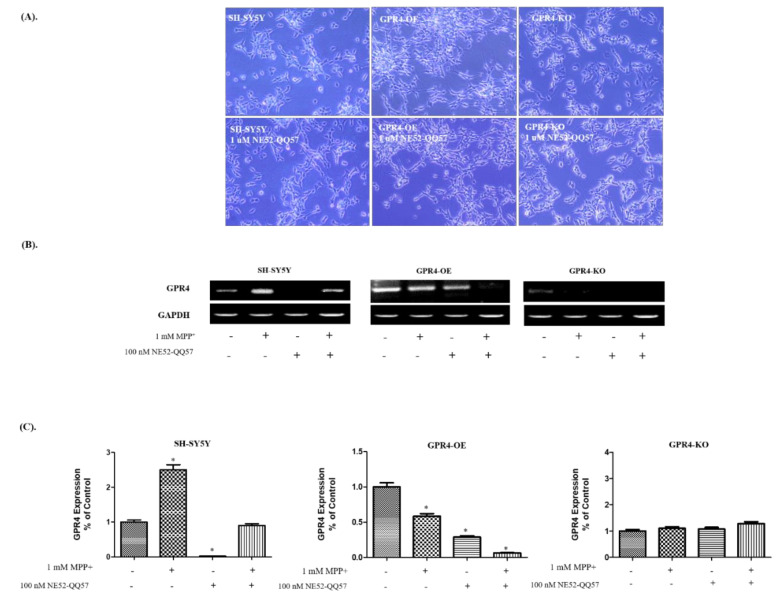
The effect of GPR4 antagonist, NE52-QQ57, on the cellular morphology and on GPR4 mRNA expression in MPP^+^-treated SH-SY5Y cells that were stably GPR4-OE or GPR4-KO. 24 h serum-starved SH-SY5Y cells were treated for 1 h with NE52-QQ57 followed by either a 24 h incubation or MPP^+^ (1 mM) stimulation for 24 h in serum-free culture media. (**A**) The cell viability and morphology of SH-SY5Y, GPR4-OE, and GPR4-KO cells that were pre-treated for 1 h with NE52-QQ57. (**B**) An RT-PCR highlighting the mRNA expression of GPR4 and GAPDH in SH-SY5Y, GPR4-OE, and GPR4-KO cells pre-treated for 1 h with NE52-QQ57. (**C**) A semi-quantification of GPR4 mRNA expression relative to GAPDH. This semi-quantification of the respective mRNA expression levels was performed on ImageJ software; GAPDH was utilised as an internal control. Mean ± SEM (*n* = 3) was employed to express the data. Tukey’s multiple comparison test was performed using a one-way ANOVA. Each * *p* < 0.05 refers to the sample concentration compared with the same group of non-treated cells.

To investigate the effect of the pharmacological inhibition of GPR4, we adopted a GPR4 antagonist, NE52-QQ57. At the physiological pH, NE 52-QQ57 has been reported to effectively block the cAMP that is released by GPR4 activation (IC_50_ 26.8 nM) in HEK293 cells [3]. In this study, the impact of GPR4 antagonist, NE52-QQ57, on the cellular morphology and mRNA expression of GPR4 in MPP^+^- (1 mM) treated, 24 h serum-starved SH-SY5Y cells was assessed (Figure 4). The SH-SY5Y cells were treated with NE52-QQ57 (1 µM) at pH 7.4 and incubated for 24 h in serum-free culture media, to evaluate the effect of NE52-QQ57 on cell morphology and viability. However, no morphological alteration or cellular toxicity was observed in the control SH-SY5Y, GPR4-OE, or GPR4-KO cells (Figure 4A).

For an RT-PCR, RNA was isolated from the cells pre-treated with NE52-QQ57 (1 µM) at pH 7.4; this was incubated for an additional 24 h in serum-free media, with or without MPP^+^ (1 mM) stimulation. The resulting levels of GPR4 mRNA expression illustrated that the NE52-QQ5 (100 nM) had effectively blocked the expression of GPR4 on the control SH-SY5Y and the GPR4-OE cells, similar to genetically GPR4-KO cells (Figure 4B).

A semi-quantitative analysis (Figure 4C) of the RT-PCR bands demonstrated that the NE52-QQ5 significantly reduced GPR4 expression in the SH-SY5Y (0.02 ± 0.001 folds) and the GPR4-OE cells (0.29 ± 0.02 folds), compared with the nontreated SH-SY5Y and GPR4-OE cells, respectively. Moreover, treatment with NE52-QQ5 (100 nM) decreased GPR4 mRNA expression in the MPP^+^ (1 mM) treated SH-SY5Y (0.9 ± 0.06 folds) and GPR4-OE cells (0.06 ± 0.004 folds), compared with the MPP^+^-stimulated SH-SY5Y and GPR4-OE cells, respectively.

### 2.5. Knockout of GPR4 Decreases the Bax/Bcl-2 Protein Ratio and the Cleavage of PARP Expression in Neurotoxin-Stimulated SH-SY5Y Cells

**Figure 5:**
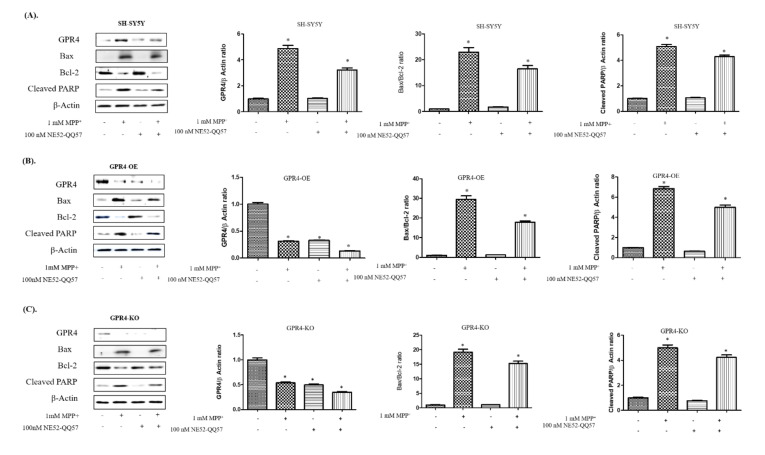
The effect of GPR4 antagonist, NE52-QQ57, on the GPR4 and pro-apoptotic protein expressions in MPP^+^-treated SH-SY5Y cells that were stably GPR4-OE or GPR4-KO. 24 h serum-starved SH-SY5Y cells were pre-treated for 1 h with NE52-QQ57 (100 µM); this was followed by MPP^+^ (1 mM) stimulation for 24 h in serum-free culture media. (**A**) An immunoblot and semi-quantification of the respective protein expressions of GPR4, Bax, Bcl-2, Cleaved PARP, and β-Actin in SH-SY5Y cells. (**B**) An immunoblot and semi-quantification of the respective protein expressions of GPR4, Bax, Bcl-2, cleaved PARP, and β-Actin in GPR4-OE cells. (**C**) An immunoblot and semi-quantification of the respective protein expressions of GPR4, Bax, Bcl-2, cleaved PARP, and β-Actin in GPR4-KO cells. β-Actin was utilised as an internal control. Mean ± SEM (*n* = 3) was employed to express the data. Tukey’s multiple comparison test was performed using a one-way ANOVA. Each * *p* < 0.05 refers to the sample concentration compared with the same group of non-treated cells.

MPP^+^-treated SH-SY5Y cells were assessed with immunoblotting to evaluate the effect of GPR4 antagonist, NE52-QQ57, on GPR4; the pro-apoptotic proteins, Bax and Bcl-2; and cleaved PARP expression (Figure 5). The SH-SY5Y cells were pre-treated with NE52-QQ57 (100 nM) at pH 7.4. This was followed by 24 h incubation, or MPP^+^ (1 mM) stimulation for 24 h in serum-free media.

For the control SH-SY5Y cells, MPP^+^ stimulation significantly increased the Bax/Bcl-2 ratio (22.94 ± 2.02 folds) and the cleaved PARP (5.09 ± 0.18 folds), in comparison with the non-treated control SH-SY5Y cells. Pre-treatment with the NE52-QQ57 (100 nM) significantly lowered the GPR4 expression (3.21 ± 0.18 folds), Bax/Bcl-2 ratio (16.47 ± 1.45 folds), and cleavage of PARP (4.29 ± 0.15 folds) in the MPP^+^-stimulated cells, in comparison with the SH-SY5Y cells that were only MPP^+^-treated (Figure 5A). For GPR4-OE cells, MPP^+^ stimulation significantly increased the Bax/Bcl-2 ratio (29.49 ± 2.06 folds) and the cleaved PARP (6.85 ± 0.23 folds), in comparison with the non-treated GPR4-OE cells. Similar to the RT-PCR results, pre-treatment with NE52-QQ57 (100 nM) significantly lowered the GPR4 expression (0.13 ± 0.003 folds), Bax/Bcl-2 ratio (17.81 ± 0.86 folds), and cleavage of PARP (4.98 ± 0.26 folds) in the MPP^+^- and NE52-QQ57-treated cells, in comparison with the GPR4-OE cells that were only treated with MPP^+^ (Figure 5B). In the GPR4-KO cells, MPP^+^ increased the Bax/Bcl-2 ratio (19.15 ± 1.45 folds) and cleaved PARP (4.99 ± 0.27 folds), in comparison with non-treated GPR4-KO cells. In contrast, MPP^+^-stimulated GPR4-KO cells that were pre-treated with NE52-QQ57 (100 nM) demonstrated only a minor increase in Bax/Bcl-2 ratio (15.24 ± 0.91 folds) and cleavage of PARP (4.23 ± 0.22 folds), in comparison with GPR4-KO cells that were only MPP^+^-treated (Figure 5C). Overall, the GPR4-KO cells displayed a lesser increase in the Bax/Bcl-2 ratio (19.15 ± 1.45 folds) and a decrease in the PARP cleavage (4.99 ± 0.27 folds), in comparison with both the MPP^+^-stimulated SHSY-5Y (Bax/Bcl-2, 22.94 ± 2.02 folds; cleaved PARP, 5.09 ± 0.18 folds) and GPR4-OE cells (Bax/Bcl-2, 29.49 ± 2.06 folds; cleaved PARP, 6.85 ± 0.23 folds). Thus, less apoptotic cell death was induced by MPP^+^.

### 2.6. Knockout of GPR4 Decreases the Caspase-3 Activity and Lowers the ROS Generation in Neurotoxin-Stimulated SH-SY5Y Cells

**Figure 6:**
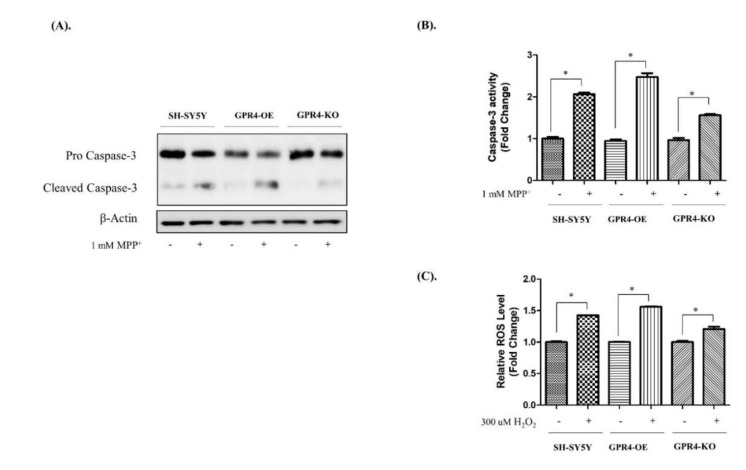
The Caspase-3 activity and intracellular ROS generation in MPP^+^- and H_2_O_2_-treated SH-SY5Y cells that were stably GPR4-OE or GPR4-KO. 24 h serum-starved SH-SY5Y cells were treated with MPP^+^ (1 mM) for 24 h in serum-free culture media for an immunoblot and a caspase activity assay. SH-SY5Y, GPR4-OE, and GPR4-KO cells were treated with H_2_O_2_ (300 µM) for 1 h in serum-free culture media for a 2′,7′-dichlorofluorescein diacetate (DCFDA) assay. (**A**) An immunoblot of the pro caspase & cleaved Caspase-3 and β-Actin. (**B**) Caspase-3 activity was measured using a colorimetric assay kit (Sigma, CAS No. CASP-3-C) in MPP^+^-induced apoptotic cells. (**C**) The relative intracellular ROS level after 1 h of H_2_O_2_ (300 µM) treatment of 24 h serum-starved SH-SY5Y, GPR4-OE, and GPR4-KO cells. β-Actin was utilised as an internal control. Mean ± SEM (*n* = 3) was employed to express the data. Tukey’s multiple comparison test was performed using a one-way ANOVA. Each * *p* < 0.05 refers to the sample concentration compared with the same group of non-treated cells.

Caspases are important factors that trigger apoptosis. Caspase-3, in particular, is a crucial biomarker and executor of neuronal apoptosis [31]. To evaluate Caspase-3 activity, immunoblotting and a caspase activity assay were performed (Figure 6). SH-SY5Y cells were treated with MPP^+^ (1 mM) for 24 h in serum-free media, while cell lysates were analysed through a western blot and a caspase activity assay. MPP^+^-treated GPR4-OE cells demonstrated a significant increase in their cleaved Caspase-3 protein levels, whereas knockout of GPR4 prevented an MPP^+^ stimulated increase in the level of cleaved Caspase-3 (Figure 6A).

The Caspase-3 activity assay results were in complete agreement with the Caspase-3 activity demonstrated in the immunoblot data (Figure 6B). MPP^+^-treated GPR4-KO cells presented a lower level of caspase activity (1.55 ± 0.03 folds) than the MPP^+^-treated SHSY-5Y (2.06 ± 0.04 folds) and GPR4-OE cells (2.47 ± 0.011 folds; Figure 6B).

We evaluated the effects of GPR4 overexpression and knockout on H_2_O_2_-induced intracellular ROS generation in SH-SY5Y cells [19]. In our study, the protective effect of the knockout of GPR4 against H_2_O_2_ resulted in lower intracellular ROS levels measured in the SH-SY5Y cells. DCFDA, a fluorescent dye that in the presence of ROS is oxidised to fluorescent DCF, was utilised for the detection of intracellular ROS levels. Treatment of the SH-SY5Y cells with H_2_O_2_ (300 µM) for 1 h led to a marked increase in their intracellular ROS levels. In H_2_O_2_-treated GPR4-OE cells, the level of intracellular ROS generation was 1.56 ± 0.01 folds higher than that of the non-treated GPR4-OE cells, whereas the H_2_O_2_-treated GPR4-KO cells demonstrated a lower level of ROS generation (1.20 ± 0.04 folds) in comparison with both the H_2_O_2_-treated control SH-SY5Y (1.42 ± 0.001 folds) and the H_2_O_2_-treated GPR4-OE cells (1.56 ± 0.01 folds; Figure 6B).

### 2.7. Knockout of GPR4 Increases the Mitochondrial Membrane Potential (MMP) in Neurotoxin-Stimulated SH-SY5Y Cells

Excess intracellular ROS leads to swelling of the mitochondrial matrix and rupture of the outer membrane, which opens up the mitochondrial permeability transition pores (mPTPs). As a result, the mitochondrial membrane potential (MMP) is disrupted and mitochondrial oxidative stress-mediated apoptosis is initiated [4].

In this study, to measure the MMP, 24 h serum-starved SH-SY5Y cells were treated with MPP^+^ (1 mM) for 24 h in serum-free culture media. MMP was then determined through a JC-10 fluorescence quantitative assay. Similarly, in a separate experiment in a 6-well plate, cells were utilised for JC-10 fluorescence microscopy to visualise the red and green fluorescence.

**Figure 7:**
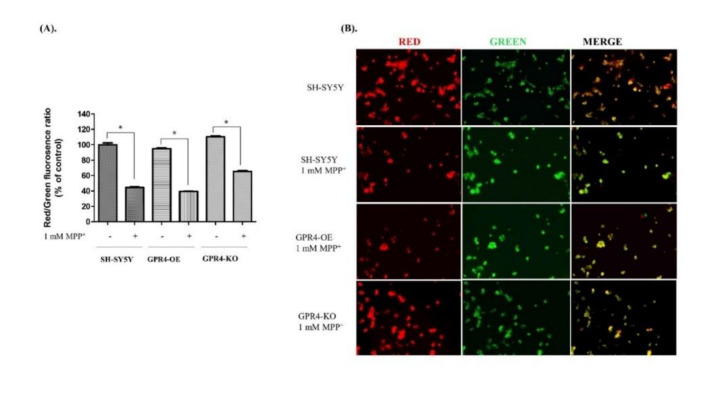
The measurement of mitochondrial membrane potential (MMP) in MPP^+^-treated SH-SY5Y cells that were stably GPR4-OE or GPR4-GPR4-KO. 24 h serum-starved SH-SY5Y cells were treated with MPP^+^ (1 mM) for 24 h in serum-free culture media; a JC-10 fluorescence quantitative assay (according to the manufacturer’s instructions) and fluorescence microscopy were then employed to measure their MMP. (**A**) The percentage ratios of J-aggregates (red) and J-monomers (green). (**B**) MMP changes were monitored with a JC-10 dye that is detectable through fluorescence microscopy. Mean ± SEM (*n* = 3) was employed to express the data. Tukey’s multiple comparison test was performed using a one-way ANOVA. Each * *p* < 0.05 refers to the sample concentration compared with the same group of non-treated cells.

Aggregated JC-10 is an indicator of MMP; the greater the ratio of red/green fluorescence, the higher the level of MMP. In a quantitative JC-10 fluorescence microplate assay, the MPP^+^-treated SHSY-5Y cells presented a lower red/green fluorescence ratio (44.75 ± 0.82%) than the untreated SH-SY5Y cells (Figure 7A). The MPP^+^-treated GPR4-OE cells, meanwhile, displayed the lowest red/green fluorescence ratio of all the samples (39.44 ± 0.39%), indicating a loss of MMP. In contrast, GPR4 knockout prevented the loss of MMP for the MPP^+^-treated GPR4-KO cells, as indicated by a higher red/green fluorescence ratio (65.44 ± 0.99%). Therefore, the MPP^+^-stimulated GPR4-KO cells demonstrated a higher level of MMP than either the MPP^+^-stimulated SH-SY5Y (44.75 ± 0.82%) or the GPR-OE (39.44 ± 0.39%) cells.

In this study, the aggregated JC-10 created red fluorescence in the polarised mitochondrial membrane. When the MMP collapsed in apoptotic cells, the JC-10 retained its monomeric form, which is characterised by green fluorescence. An increase in the red/green fluorescence intensity ratio indicated intact mitochondria. In a separate experiment, to visualise the JC-10 fluorescence dye in the MPP^+^-treated cells, JC-10 fluorescence microscopy was employed. In the control SHSY-5Y cells, both red and green fluorescence was observed, with a high level of red fluorescence and low level of green fluorescence. In contrast, the level of green fluorescence was higher and the red fluorescence remarkably lower in both the MPP^+^-treated SHSY-5Y and GPR4-OE cells, when compared with the control SHSY-5Y cells. In the MPP^+^-treated GPR4-KO cells, the red fluorescence was restored close to that of the control SHSY-5Y cells, while the level of green fluorescence was decreased (Figure 7B). These results suggest that GPR4 knockout restores the MPP^+^-induced a loss of MMP in dopaminergic neurons.

### 2.8. Knockout of GPR4 Decreases the Intracellular Calcium in Neurotoxin-Stimulated SH-SY5Y Cells

**Figure 8:**
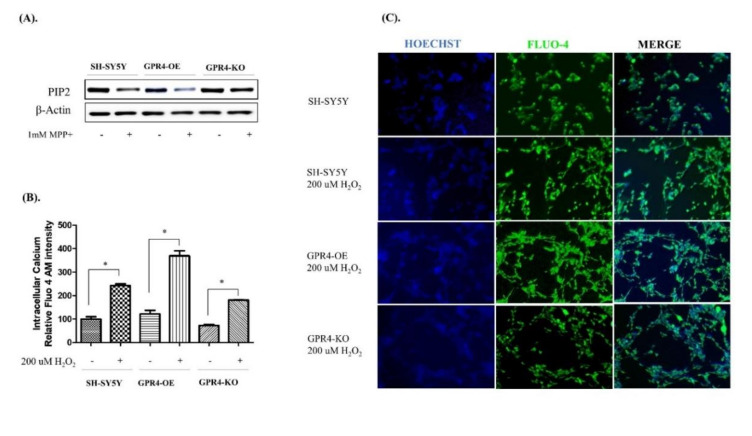
Phosphatidylinositol biphosphate (PIP_2_) calcium signalling and intracellular calcium levels in MPP^+^-treated SH-SY5Y cells that were stably GPR4-OE or GPR4-KO. 24 h serum-starved SH-SY5Y cells were treated with MPP^+^ (1 mM) for 24 h in serum-free culture media for the purpose of immunoblotting. The SH-SY5Y cells were then treated with H_2_O_2_ (200 µM) for 2 h 30 min in serum-free culture media and subjected to a Fluo-4 AM fluorescence assay and fluorescence microscopy. Detection of the Fluo-4 AM fluorescence intensity and related imaging were carried out according to the manufacturer’s instructions. Cells were counter-stained with Hoechst dye. (**A**) An immunoblot of the PIP_2_ and β-Actin. (**B**) A quantitative analysis of the Fluo-4 AM fluorescence intensity in H_2_O_2_- (200 µM) treated cells. (**C**) Fluo-4 AM calcium imaging of the intracellular calcium level. Mean ± SEM (*n* = 3) was employed to express the data. Tukey’s multiple comparison test was performed using a one-way ANOVA. Each * *p* < 0.05 refers to the sample concentration compared with the same group of non-treated cells.

Increases in intracellular Ca^2+^ in association with MPP^+^- or H_2_O_2_-mediated apoptotic cell death have been previously reported [32]. Several studies have suggested that an increase in the intracellular Ca^2+^ released from the ER store by the inositol trisphosphate receptor (IP_3_R) is directly responsible for mitochondrial Ca^2+^ overload [33,34]. However, the exact mechanism by which MPP^+^ or H_2_O_2_ stimulation increases the intracellular calcium is not clearly understood. Interestingly, several studies have demonstrated that H_2_O_2_-/MPP^+^-mediated mitochondrial oxidative stress is associated with an intracellular Ca^2+^ spike, which increases the Bax/Bcl-2 ratio, the release of cytochrome C, mitochondrial depolarisation, and the Caspase-3 activity in neuronal cells [19,32]. Previous reports have suggested that many G protein coupled-receptors (GPCRs), such as GPR4, which releases G_βγ_ and activates G_i_, are capable of Ca^2+^ signalling. Few GPCRs, however, harness G_βγ_-dependent activation of PLC_β_ to release ER-stored Ca^2+^ into the cytoplasm through PIP_2_ degradation [35,36]. In this study, the MPP^+^-treated GPR4-OE cells demonstrated an increased proteolytic degradation of PIP_2_, in comparison with the SH-SY5Y cells treated with MPP^+^. Contrastingly, the MPP^+^-stimulated GPR4-KO cells presented a particularly low degradation of PIP_2_ compared with both the MPP^+^-stimulated SH-SY5Y and GPR4-OE cells (Figure 8A).

To evaluate whether GPR4 overexpression increased intracellular calcium through G_βγ_ modulation of the PLC_β_-PIP_2_ pathway, SH-SY5Y cells were treated with MPP^+^ (1 mM) for 24 h in serum-free media. Cell lysates were analysed through western blotting to determine the degradation of PIP_2_. The SH-SY5Y cells were treated with H_2_O_2_ (200 µM) for 2 h 30 min to determine their relative intracellular Ca^2+^, utilising a Fluo-4 AM calcium indicator in a fluorescence microplate assay. Similarly, in a separate 6-well plate, cells stained with a Fluo-4 AM calcium indicator were observed under a fluorescence microscope.

The quantitative analysis of intracellular Ca^2+^, as indicated by the Fluo-4 AM microplate assay, found levels of intracellular Ca^2+^ for the H_2_O_2_-treated GPR4-OE cells that were 369.58 ± 24.75% higher than those for the non-treated GPR4-OE cells. In contrast, the H_2_O_2_-treated GPR4-KO cells presented significantly lower levels of intracellular Ca^2+^ (181.28 ± 0.85%), in comparison with the H_2_O_2_-treated SH-SY5Y (243.25 ± 7.81%) and H_2_O_2_-treated GPR4-OE cells (369.58 ± 24.75%; Figure 8B).

In a separate experiment to visualise intracellular Ca^2+^ levels in the H_2_O_2_-treated cells, Fluo-4 AM fluorescence microscopy was employed. This round of microscopy demonstrated similar results to those obtained from the quantitative microplate assay. H_2_O_2_-treated GPR4-OE cells displayed the highest levels of green Fluo-4 AM fluorescence, while H_2_O_2_-treated GPR4-KO cells produced lower levels of green Fluo-4 AM fluorescence than both the H_2_O_2_-treated SH-SY5Y and GPR4-KO cells (Figure 8C). Overall, these data suggest that the increase in intracellular calcium associated with H_2_O_2_- or MPP^+^-mediated mitochondrial oxidative stress is exaggerated by GPR4 overexpression, whereas GPR4 knockout prevents an increase in intracellular Ca^2+^ through the decrease of PIP_2_ degradation, and thus restricts the release of Ca^2+^ from the ER by preventing the degradation of PIP_2_. Therefore, GPR4-PLC_β_-PIP_2_ signalling may act as a key factor through which GPR4 increases intracellular calcium and potentiates mitochondrial oxidative stress-mediated apoptosis.

## 3. Discussion

In this study, we investigated the roles of GPR4 overexpression, pharmacological inhibition, and genetic knockout in the mitochondrial oxidative stress-induced apoptotic cell death that is associated with PD. Although many studies have reported the activation of GPR4 at the physiological pH range (7.0–7.4), overexpression of GPR4 showed relatively high GPR4 activity at neutral pH 7.4 [37]. In transiently GPR4-overexpressing HEK293 cells, GPR4 is inactive at pHs higher than 8.0, whereas it is highly active at the physiological pH, 7.4, and substantially less active at pHs down to 6.8 (plausible in the range of physiological acidification) [38]. The pH sensitivity of GPR4 has been reported to vary for different cells, though potentially due to the methods employed in different laboratories [25]. In the natively GPR4-expressing cell, HUVEC, pHs from 7.4 to 7.0 have been shown to result in a 1.5-fold activation of GPR4 [25]. In this study, we found an increase in GPR4 mRNA expression at pH 7.4 in both SH-SY5Y and stably GPR4-OE cells in serum-starved media (data not added). A very slight increase in the expression of GPR4 was observed at pH 6.4. Therefore, to maintain consistency, we conducted all the experiments at a pH ~7.4. This was also the pH of the culture media that we employed.

Human-derived neuroblastoma SH-SY5Y cells are widely used in neuroscientific research as an in vitro model for the investigation of neuronal differentiation and neuroprotective events. Stimulation with several neurotoxins, such as MPP^+^, MPTP, rotenone, 6-OHDA, and H_2_O_2_, has been utilised to induce oxidative stress-mediated apoptotic death, thereby mimicking neurodegenerative diseases, including PD and aging [39,40,41]. To determine the final concentration of H_2_O_2_ and MPP^+^, SH-SY5Y cells were treated with H_2_O_2_ at different concentrations, ranging from 75 μM to 125 μM, for 24 h, as well as with MPP^+^, ranging from 250 µM to 1 mM. H_2_O_2_ and MPP^+^ both decreased the cell viability in a concentration-dependent manner, with optimum cytotoxicity being observed at concentrations of 125 μM for H_2_O_2_ and 1 mM for MPP^+^; these concentrations were selected for further experiments to determine their cytotoxicity in the serum-free SH-SY5Y cell line. In our study, GPR4 mRNA and protein expressions were increased in a time-dependent manner for 24 h in both MPP^+^- and H_2_O_2_-treated SH-SY5Y cells. Hence, GPR4 is directly linked with MPP^+^- and H_2_O_2_-induced apoptotic cell death.

Both the pro-apoptotic protein, Bax, and the anti-apoptotic protein, Bcl-2, are members of the Bcl-2 family and are directly involved in apoptotic cell death. The balance between these two proteins of the Bcl-2 family, or an increase in the Bax/Bcl-2 ratio, indicates the early phases of an apoptotic cascade [29,30]. Significant increases in ROS, or the Bax/Bcl-2 ratio, result in the collapse of the mitochondrial membrane potential, the release of cytochrome C, the activation of Caspase-3, the cleavage of PARP, and, subsequently, apoptotic cell death [6,7]. Both MPP^+^- and H_2_O_2_-induced apoptotic cell deaths bear the characteristic hallmarks of an increase in the Bax/Bcl-2 ratio, the release of cytochrome-C, and the activation of the proteolytic enzyme, Caspase-3, which cleaves PARP and induces apoptotic cell death [7,8]. In our study, the overexpression of GPR4 in SH-SY5Y cells significantly increased the effect of either MPP^+^ or H_2_O_2_ and increased the Bax/Bcl-2 ratio, as was seen in both the immunoblot and RT-PCR. As a result, this significantly increased the protein level of the cleaved Caspase-3, the Caspase-3 mediated cleavage of PARP, and the Caspase-3 activity. On the contrary, the CRISPR/Cas9 knockout of GPR4 was found to result in a lesser increase in the Bax/Bcl-2 mRNA and protein ratio in both MPP^+^- and H_2_O_2_-treated cells. Knockout of GPR4 was also shown to reduce the cleavage of PARP after MPP^+^ treatment. NE52-QQ57, a selective antagonist of GPR4, demonstrated a similar level of the inhibition of GPR4 expression, as was determined through both our immunoblots and RT-PCR.

We further investigated the effect of GPR4 on mitochondrial oxidative stress-induced increases in intracellular ROS generation and MMP. Surprisingly, GPR4-OE was found to significantly increase tricellular ROS generation in SH-SY5Y cells, whereas GPR4-KO generated a lower level of intracellular ROS accumulation, after a high concentration of H_2_O_2_ treatment. Similarly, through both a JC-10 assay and fluorescence microscopy, the knockout of GPR4 was found to decrease mitochondrial membrane depolarisation. In JC-10-tagged fluorescence microscopy, knockout of GPR4 was seen to prevent MPP^+^ stimulated decrease red fluorescence and increase green fluorescence. The latter was highly increased in the case of GPR4-OE as membrane depolarisation occurs 24 h after MPP^+^ treatment.

Besides mitochondrial dysfunction, abnormal protein aggregation and dysregulated Ca^2+^ homeostasis are other factors that may be involved in the neurodegeneration observed in individuals with PD [42]. Recent findings suggest that increases in cytosolic Ca^2+^ occur at both early and late stages of the apoptotic pathway. In both cases, ER Ca^2+^ channels are linked with the release of Ca^2+^ to the cytoplasm [33,34]. However, the exact mechanism by which intracellular Ca^2+^ modulates mitochondrial oxidative stress-mediated apoptosis remains elusive. Many studies have suggested that MPP^+^- and H_2_O_2_-induced apoptosis are associated with an increase in intracellular calcium levels [43]. For example, Sing et al. (2016) demonstrated that the administration of Nimodipine, an L-type calcium channel blocker, protected from MPTP-induced dopaminergic neuronal death in an animal model of PD. More importantly, providing evidence for Nimodipine as a means to improve mitochondrial integrity and function. In the study, Nimodipine attenuated the MPTP-induced loss of tyrosine hydroxylase-positive dopaminergic neurons in the SN. It also improved mitochondrial oxygen consumption and inhibited ROS production, as well as improving mitochondrial integrity and function in striatal mitochondria [43]. These findings provide evidence in support of the notion that calcium signalling is linked with neurotoxin-induced mitochondrial dysfunction and neurodegeneration. GPR4 is a G_s_-coupled receptor that signals through adenylate cyclase and also via G proteins G_13_ and G_q/11_. GPR4 is well known for its ability to recognise phospholipase C β (PLC_β_) as its canonical target [44]. G_q_ class α subunits, or G_βγ_ released by GPCR, activate Ca^2+^ signalling through G_βγ_-dependent activation of PLC_β_. Upon activation, PLC_β_ hydrolyses PIP_2_ to generate IP_3_. IP_3_ binds to the ER-resident IP_3_ receptors, which act as Ca^2+^ release channels to release ER-stored Ca^2+^ into the cytoplasm [35]. In this study, overexpression of GPR4 significantly increased the intracellular calcium level in both MPP^+^- and H_2_O_2_-treated cells (MPP^+^ data not given), whereas knockout generated very little change in the intracellular calcium. These findings were also observed in our study when employing the Fluo-4 AM indicator. To determine how GPR4 can modulate the intracellular calcium level, we investigated GPCR-mediated calcium signalling. We found that GPR4 knockout decreases the breakdown of PIP_2_, which is a critical step in Ca^2+^ release from the ER to the cytoplasm. Therefore, decreased intracellular Ca^2+^ may be responsible for GPR4-mediated neuroprotection against MPP^+^- or H_2_O_2_-induced apoptotic cell death. Our study, for the first time, demonstrated that the knockout of GPR4 protects SH-SY5Y cells from both MPP^+^- and H_2_O_2_-stimulated mitochondrial apoptotic cell death, in association with a decrease in intracellular Ca^2+^.

In summary, our study suggests that overexpression of GPR4 potentiates neurotoxin-induced mitochondrial oxidative stress, whereas a knockout or pharmacological inhibition of GPR4 improves the neurotoxin-induced, caspase-dependent mitochondrial apoptosis of dopaminergic neuronal cells. This study has also found that GPR4 can increase intracellular Ca^2+^ through the degradation of PIP_2_. Further investigation is required to determine how GPR4-mediated calcium signalling can mitigate the neuronal cell death seen in neurodegenerative disorders, including PD.

## 4. Materials and Methods

### 4.1. Reagents and Antibodies

1-methyl-4-phenylpyridinium ion MPP^+^, H_2_O_2_, and 3-(3,4-dimehylthiazol-2-yl)-2,5-diphenyl-tetrazolium bromide (MTT) were obtained from Sigma-Aldrich (St. Louis, MO, USA). 96-well tissue culture plates, along with six-well and 100 mm culture dishes, were obtained from Nunc Inc. (North Aurora Road, Naperville, IL, USA). Foetal bovine serum (FBS) and Dulbecco’s modified Eagle’s medium (DMEM/F12) were purchased from Gibco-BRL Technologies (Gaithersburg, MD, USA). RIPA buffer (10×) was purchased from Millipore (Milford, MA, USA). Tween 80 was obtained from Calbiochem (Gibbstown, NJ, USA). All other chemicals utilised in this research were of analytical grade and were purchased, unless otherwise noted, from Sigma-Aldrich.

### 4.2. Cell Culture and Transfection

The human dopaminergic neuroblastoma SH-SY5Y cell line was acquired from the American Type Culture Collection (ATCC; Manassas, VA, USA). SH-SY5Y cells were cultured in DMEM/F12 with or without phenol red and HEPES, supplemented with 100 U/mL penicillin/streptomycin and 10% (v/v) inactivated foetal bovine serum. The SH-SY5Y cells were maintained in a 5% CO_2_ and 95% humidified air incubator at 37 °C for the time indicated in the experiments. MPP^+^ and H_2_O_2_ were dissolved in three-times distilled water (3DW).

To overexpress and knockout the human GPR4 gene, a GPR4 lentiviral vector (CMV; pLenti-GIII-CMV-C-term-HA) and a GPR4 sgRNA CRISPR/Cas9 lentiviral vector (pLenti-U6-sgRNA-SFFV-Cas9-2A-Puro) were designed, to generate stable GPR4-overexpressing (GPR4-OE) and stable GPR4-knockout (GPR4-KO) SH-SY5Y lines. Briefly, the SH-SY5Y cells were transferred into 60 mm plates at a density of 5 × 10^4^ cells/mL to achieve ~70% confluence at the time of transfection. The cells were transfected using the Lipofectamine^®^ 3000 transfection reagent (ThermoFisher, Langenselbold Germany; #L3000015), according to the manufacturer’s protocol. Both the GPR4-overexpression and knockout-silencing genes were designed to carry puromycin-resistance genes and were produced using the Lentivector Expression System (Applied Biological Materials Inc. (ABM), Canada). Stable single clones were selected following 3–5 weeks of puromycin treatment (1 μg/μL). GPR4 overexpression and knockout in the stably infected clones were assessed through RT-PCR and western blotting. A sequence analysis of the GPR4 insert was also employed.

### 4.3. Measurement of Cell Viability

The cytotoxicity of the MPP^+^- and H_2_O_2_-treated SH-SY5Y cells was measured with an MTT assay, involving the reduction of formazan crystals [41]. SH-SY5Y cells (2.2 × 10^4^ cells/mL) were pre-treated in 24-well plates with NE 52-QQ57 (100 nM) and left in serum-free cell culture media for 1 h; this was followed by stimulation with or without MPP^+^ (1 mM) or H_2_O_2_ for 24 h. After MPP^+^ or H_2_O_2_ stimulation, the medium was replaced with 0.5 mg/mL MTT solution, before the plates were incubated for 3 h at 37 °C. The supernatant was carefully removed and the formazan crystals were dissolved in dimethyl sulfoxide (DMSO) by gentle shaking for 10 min. A microplate reader (Molecular device, Sunnyvale, CA, USA) was utilised to measure the absorbance at 550 nm.

### 4.4. Total RNA Isolation for RT-PCR

SH-SY5Y cells (2.2 × 10^4^ cells/mL) were pre-treated in 60 mm cell culture dishes with NE 52-QQ57 (100 nM), then left in serum-free cell culture media for 1 h, followed by stimulation with or without MPP^+^ (1 mM) or H_2_O_2_ for 18 h, once again in serum-free media. TRIzol (Invitrogen; Burlington, ON, Canada) was employed to extract the total RNA from the cells. 2.5 µg total RNA from each group was reverse-transcribed using a first-strand cDNA synthesis kit (Invitrogen). The following primers were utilised for PCR: GPR4 sense, 5′-CCGTTGTCAAGACCGGGG-3′; GPR4 anti-sense, 5′-TCCTAGGACCCCCAGAAAGCA-3′; Bax sense, 5′-CACCAAGGTGCCGGAACTGA-3′; Bax anti-sense, 5′-AATGCCCATGTCCCCCAATC-3′; Bcl-2 sense, 5′-ACGACTTCTCCCGCCGCTAC-3′; Bcl-2 anti-sense, 5′-CCCAGCCTCCGTTATCCTGG-3′; GAPDH sense, 5′-GCAGTGGCAAAGTGGAGATTG-3′; and GAPDH anti-sense, 5′-TGCAGGATGCATTGCTGACA-3′. Then, adopting the previously mentioned primers, the cDNA was amplified through PCR [45]. GAPDH was employed as an internal control to evaluate the relative levels of expression of other genes. PCR products were analysed on 1.0–1.2% agarose gels stained with GelRed (Sigma-Aldrich; St. Louis, MO, USA). The gels were photographed and, utilising ImageJ (NIH) software, the pixel intensity for each band in the photographs was measured and normalised to the band intensity of the GAPDH mRNA, to quantify its relative expression.

### 4.5. Immunoblot Analysis

SH-SY5Y cells (2.2 × 10^4^ cells/mL) were pre-treated in 60 mm cell culture dishes with NE 52-QQ57 (100 nM) and left in serum-free media for 1 h. They were then stimulated with or without MPP^+^ (1 mM) or H_2_O_2_ for 24 h, again in a serum-free media. Next, the cells were washed two times with PBS and lysed for 10 min at 4 °C using an RIPA lysis buffer (with protease and phosphatase inhibitors). Supernatants were collected for further investigation after the cell lysates were centrifuged at 14,000 rpm, at 4 °C. The protein concentration of each sample was measured and normalised using a DC Protein Assay kit (Bio-Rad). Equal amounts of proteins (20–30 μg) were loaded and separated electrophoretically in 8, 10, and 12% sodium dodecyl sulphate-polyacrylamide gels; these were then transferred to polyvinylidene difluoride membranes (Millipore; Bedford, MA, USA). The membranes were incubated overnight at 4 °C, with corresponding primary antibodies, GPR4 (1:500) from Novus Biologicals (Centennial, CO, USA), BCL-2 (1:1000), Caspase-3 (1:1000), cleaved Caspase-3 (1:1000), cleaved PARP (1:1000) from Santa Cruz Biotechnology (Santa Cruz, CA, USA), Bax (1:1000) from Cell Signaling Co. (Boston, MA, USA), PIP_2_ (1:500) from Abcam (Cambridge, United Kingdom), and β-Actin (1:2000) from Sigma-Aldrich (St. Louis, MO, USA), followed by 1 h incubation with horseradish peroxidase (HRP)-conjugated secondary antibodies (1:2000; Cell signalling, MA, USA). The blots were visualised using a Biorad-ECL (Bio-Rad Laboratories; Hercules, CA, USA) and photographed. Using ImageJ (NIH) software, the pixel intensity for each band in the photographs was measured and normalised to the band intensity of β-Actin, to quantify its relative expression.

### 4.6. Detection of Intracellular ROS

The ROS-sensitive fluorescent dye, 2′,7′-dichlorofluorescein diacetate (DCFDA; Sigma-Aldrich), was utilised to measure the intracellular ROS levels. SH-SY5Y cells (2.2 × 10^4^ cells/mL) were cultured in black 96-well plates in DMEM/F12 without phenol red. Then, 60–70% confluence cells were stimulated with H_2_O_2_ (300 µM) for 1 h in serum-free media and then washed twice with PBS, followed by a 30 min incubation with DCFDA (10 μM) in PBS. The cells were then rinsed with PBS twice. Finally, 200 µL PBS was added, and fluorescence was measured using 485 nm excitation and 535 nm in a fluorescence microplate reader (Molecular Device; Sunnyvale, CA, USA).

### 4.7. Assessment of Caspase-3 Activity

Caspase-3 activity was measured using a Colorimetric Caspase-3 Assay Kit (Sigma-Aldrich; St. Louis, MO, USA), as described previously [41]. The reaction mixture (total volume, 200 µL) was distributed in 96-well plates and incubated at 37 °C for 90 min. Absorbance values were measured at wavelengths of 405 nm in a Tecan Microplate Reader (Meilen; Zurich, Switzerland).

### 4.8. Assessment of Mitochondrial Membrane Potential (MMP)

A JC-10-based Mitochondrial Membrane Potential Assay Kit (Abcam; Cambridge, United Kingdom) was employed to assess MMP, while a fluorescence microscope was utilised to visualise the JC-10 staining, according to the manufacturer’s instructions. In brief, SH-SY5Y cells (2.2 × 10^4^ cells/mL) were cultured in black, 96-well plates for quantification and in 6-well plates for imaging in DMEM/F12 without phenol red. Then, 60–70% confluence cells were stimulated with MPP^+^ (1 mM) for 24 h in serum-free media. The cells were incubated with a JC-10 dye loading solution at 37 °C for 1 h and protected from the light. For the 96-well plates, their fluorescence intensities (λ_ex_ = 490/λ_em_ = 525 nm) and (λ_ex_ = 540/λ_em_ = 590 nm) the red-green fluorescence ratios were measured using a fluorescence microplate reader (Molecular Device; Sunnyvale, CA, USA), while confocal images were acquired with a Nikon Eclipse Ts2-FL diascopic and epi-fluorescence illumination microscope.

### 4.9. Detection of Intracellular Calcium

Intracellular calcium was assessed with a Fluo-4 AM dye (Abcam; Cambridge, United Kingdom) and confocal microscopy, following the manufacturer’s instructions. Fluo-4 AM was diluted in DMSO containing 2 mM probenecid and 0.02% pluronic F-127. In brief, the SH-SY5Y cells (2.2 × 10^4^ cells/mL) were cultured in black, 96-well plates for quantification and in 6-well plates for imaging in DMEM/F12 without phenol red. Then, 60–70% confluence cells were stimulated with H_2_O_2_ (200 µM) for 2 h 30 min in serum-free media. The cells were washed with PBS containing probenecid (2 mM) at room temperature. The cells were incubated with the Fluo-4 AM (2 µM) dye loading solution at 37 °C for 30 min and protected from light, then washed with PBS containing probenecid (2 mM) at room temperature for 30 min. For the 96-well plates, the fluorescence intensities (λ_ex_ = 488/λ_em_ = 515 nm) were measured using a fluorescence microplate reader (Molecular Device; Sunnyvale, CA, USA), and confocal images were acquired with a Nikon Eclipse Ts2-FL diascopic and epi-fluorescence illumination microscope.

### 4.10. Statistical Analyses

Statistical analyses were performed using GraphPad Prism software, version 5 (GraphPad, La Jolla, CA, USA). Data are expressed as means ± standard error (SEM) of at least three independent experiments. One-way analysis of variance (ANOVA) followed by Tukey’s post hoc analysis were performed to determine the significant differences between the groups. *P*-values < 0.05 were considered statistically significant.

## Abbreviations

MPTP	1-Methyl-4-Phenyl-1,2,3,6-Tetrahydropyridine
MPP^+^	1-Methyl-4-Phenylpyridinium Ion
MTT	3-(3,4-Dimehylthiazol-2-Yl)-2,5-Diphenyl-Tetrazolium Bromide
DCF-DA	7′-Dichlorofluorescein Diacetate
DMEM	Dulbecco’s Modified Eagle’s Medium
ER	Endoplasmic Reticulum
FBS	Fetal Bovine Serum
HUVEC	Human Umbilical Vein Endothelial Cells
MMP	Mitochondrial Membrane Potential
Mptp	Mitochondrial Permeability Transition Pore
PIP2	Phosphatidylinositol Biphosphate
PARP	Poly (ADP-Ribose) Polymerase
SN	Substantia Nigra
TDAG8	T-Cell Death-Associated Gene 8
OGR1	The Ovarian Cancer G Protein-Coupled Receptor 1
